# Study of the distribution and abundance of the eggs of *Aedes aegypti* and *Aedes albopictus* according to the habitat and meteorological variables, municipality of São Sebastião, São Paulo State, Brazil

**DOI:** 10.1186/1756-3305-6-321

**Published:** 2013-11-06

**Authors:** Lígia Leandro Nunes Serpa, Gisela Rita Alvarenga Monteiro Marques, Ana Paula de Lima, Júlio Cesar Voltolini, Marylene de Brito Arduino, Gerson Laurindo Barbosa, Valmir Roberto Andrade, Virgília Luna Castor de Lima

**Affiliations:** 1 Superintendência de Controle de Endemias, Praça Coronel Vitoriano, 23 Jardim Santa Clara, Centro, Taubaté, São Paulo CEP 12020-020, Brasil; 2 Universidade de Taubaté, Avenida Tiradentes, 500, Bom Conselho, Taubaté, São Paulo CEP 12030-180, Brasil; 3 Superintendência de Controle de Endemias, Rua Paula Souza, 166, Luz, São Paulo, São Paulo CEP 01027-000, Brasil; 4 Superintendência de Controle de Endemias, Rua São Carlos, 546, Vila Industrial, Campinas, São Paulo CEP 13035-420, Brasil

**Keywords:** *Aedes*, Environment, Mosquito control, Dengue, Oviposition, Meteorological factors

## Abstract

**Background:**

This study focused on the distribution and abundance of the eggs of *Aedes aegypti* and *Aedes albopictus*.

**Methods:**

Eighty ovitraps were exposed for four days of each month in peri- and intradomiciliary environments of 40 urban residences on 20 street blocks that were drawn monthly in Sebastião, SP, between February 2011 and February 2012. The monthly distribution of positive ovitrap indices (POI) and mean egg counts per trap (MET) of *Ae. aegypti* and *Ae. albopictus* were analyzed using the Kruskal-Wallis test, followed by the Dwass-Steel-Critchlow-Fligner (DSCF) test. Spearman's rank correlation coefficient and simple linear regression were used to determine the association between the meteorological variables of temperature and rainfall and the number of ovitraps with eggs and the egg count.

**Results:**

The POI and MET of *Ae. aegypti* were higher in peridomiciliary premises. A positive correlation was found between the temperature and the number of ovitraps with eggs and the egg count of this species in domestic environments. There was no difference in the POI and MET of *Ae. albopictus* between the environments. A positive correlation was found between temperature and positive ovitraps of *Ae. albopictus* in peridomiciliary premises. The POI and MET of *Ae. aegypti* were higher than those of *Ae. albopictus*.

**Conclusions:**

Peridomiciliary premises were the preferred environments for oviposition of *Ae. aegypti*. The use of ovitraps for surveillance and vector control is reiterated.

## Background

The mosquitoes responsible for the transmission of the dengue virus, *Aedes aegypti* (Linnaeus) and *Aedes albopictus* (Skuse), are considered important vectors of the arbovirosis. These species present abundance influenced by the female behavior of oviposition, as well as their temporal space distribution, which has predominant dependency on the environment and the local climate in which they occur, with female mosquitoes searching for conditions favourable to survival of progeny [1].

In Brazil, dengue fever has become one of the key public health priorities since the late 1990s because its occurrence is directly related to various factors, including the level of local infestation by the vector [2,3].

*Ae. aegypti* and *Ae. albopictus* are the only Culicidae species of the *Stegomyia* genus found in Brazil [4]. In São Paulo State in the year 2012, 87% of the municipalities exhibited co-occurrence of these mosquitoes, according to data from the Superintendence for Endemic Disease Control (Superintendência de Controle de Endemias, Sucen). The population dynamics of adult *Ae. aegypti* is highly significant in the epidemiology of the dengue infection, thus directing the scientific community’s efforts towards elucidating this aspect, which is inherent to control expectations [5,6].

The abundance of these vectors is associated with biotic and abiotic factors. According to Braks *et al*. [4], the spatial distribution and abundance of *Ae. aegypti* are related to the effects of anthropogenic changes on the environment. Conversely, the distribution of *Ae. albopictus* is more associated with the presence of vegetation in urban and rural areas, whereas its abundance is generally limited to spaces modified by human activity. Among the environmental variables, rainfall, temperature, and relative humidity are key determining factors of the presence and frequency of these species [7]. According to Focks *et al*. [8], meteorological factors affect the mosquito metabolism, oviposition activity, and consequently, the number of eggs laid by females. Such bioecological aspects may be measured in positive ovitraps and egg density, as previously shown by Azil *et al*. [7].

In the entomological surveillance and control of dengue vectors, the likelihood of arbovirus transmission among humans is high, although it will always depend on the habits and densities of the species involved. The study of practical and operational methods using ovitraps may provide such information, which is beneficial for planning and managing the vector [9]. The ovitrap [10] is a useful tool for providing spatial and temporal data and successfully monitors the impact of control measures. Ovitrap use was recommended for differentiating infestation levels between areas, making it more sensitive than the larval survey currently used, even during low levels of vector population [7,11,12].

*Ae. aegypti* mosquito control mainly occurs on peridomiciliary breeding grounds, although it is unclear whether the distribution of outbreaks only results from a small supply of intradomiciliary water containers or also results from the preference for oviposition in peridomiciliary premises [13,14].

Certain studies have revealed a higher occurrence of *Ae. albopictus* in peridomiciles [15,16] and the prevalence of *Ae. aegypti* is either indistinguishable between both environments [16-18] or even more frequent in peridomiciliary premises [19].

Mosquito behavior in the field is a key factor in the epidemiology of diseases transmitted by mosquitoes. Different patterns of habitat occupation may be observed between these species or within a given species, albeit in different regions [20]. The coastal region of São Paulo State exhibits diverse climate characteristics [21]. The municipality of São Sebastião, a north-shore tourist resort where infestation by *Ae. albopictus* began in the early 1980s and infestation by *Ae. aegypti* began in the second half of the 1990s [22], is located in this region. Its climate, according to the Center for Meteorology and Climate Research Applied to Agriculture (Centro de Pesquisas Meteorológicas e Climáticas Aplicadas à Agricultura; CEPAGRI), is tropical rainy, without a dry season and with mild temperatures throughout the year. São Sebastião has high human-population fluctuation and successive records of dengue epidemics since 2001, and the serotypes DENV 1, DENV 2, DENV 3 [23], and, more recently, DENV 4 were isolated during that period, according to the SES/SP Vigilância Epidemiológica CVE 2013). Accordingly, it is crucial to examine the infestations by *Ae. aegypti* and *Ae. albopictus* in this municipality*.* To this end, the present study aimed to characterize the distribution and abundance of these species in peri- and intradomiciliary environments and to determine the association with meteorological factors to foster improvements in vector control measures.

## Methods

### Study area

The present study was conducted in an urban area of the municipality of São Sebastião (45°21′00″W and 23°21′20″S), on the northern coast of São Paulo State, Brazil. This municipality has a territory of approximately 400 Km^2^, with a population of 73,942 inhabitants, according to data from the Brazilian Institute of Geography and Statistics, and a population density of 184.68 inhabitants/Km^2^. São Sebastião has a coastal plain terrain with an average altitude of 10 m above sea level and an average annual temperature of approximately 24°C. According to the simplified Köppen Climate Classification System (CCS) [24], São Sebastião has an Af climate, which is characterized by the tropical rainy climate that has no dry season and an average rainfall below 60 mm in the driest month. Its urban area crosses the boundaries of the plains and invades the coastal mountains, a situation that results from the intense and disordered process of occupation pressured by the growth of the fluctuating tourist and urban-resident populations, which contributes to worsening the local basic sanitation services [23].

**Figure 1:**
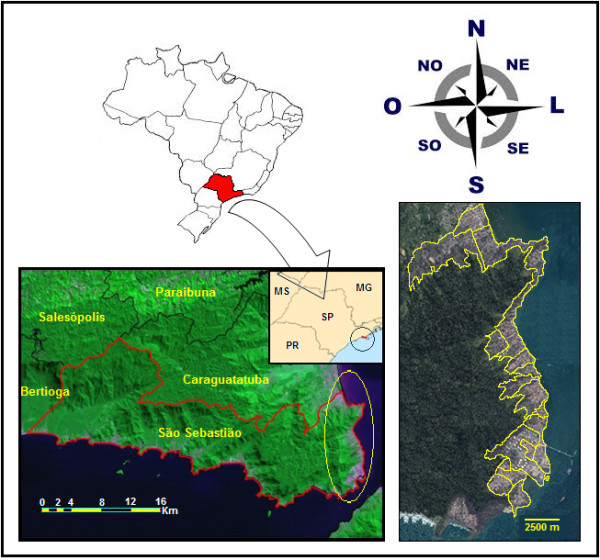
Image of the study area in the municipality of São Sebastião.

The area selected for the present study corresponds to part of the urban space and was termed Area 1 (Figure 1). This area consists of 532 street blocks, 16,833 properties, and a population of approximately 40,116 inhabitants, according to data from the Brazilian Institute of Geography and Statistics (Instituto Brasileiro de Geografia e Estatística; IBGE, 2010).

### Study design

The sample unit was the property, the street block of which was selected by systematic, random, and monthly single-stage cluster sampling with replacement [25]. Two properties were selected from each block drawn, considering the greatest distance between them. Two ovitraps were installed in these human habitations, after obtaining the owner's informed consent: one in peridomiciliary premises and the other in intradomiciliary premises, approximately 1.20 m above the floor [26]. Peridomiciliary refers to the exterior of the building, albeit limited to the immediate vicinity of the house, while intradomiciliary refers to areas within the house, under its roof.

**Figure 2:**
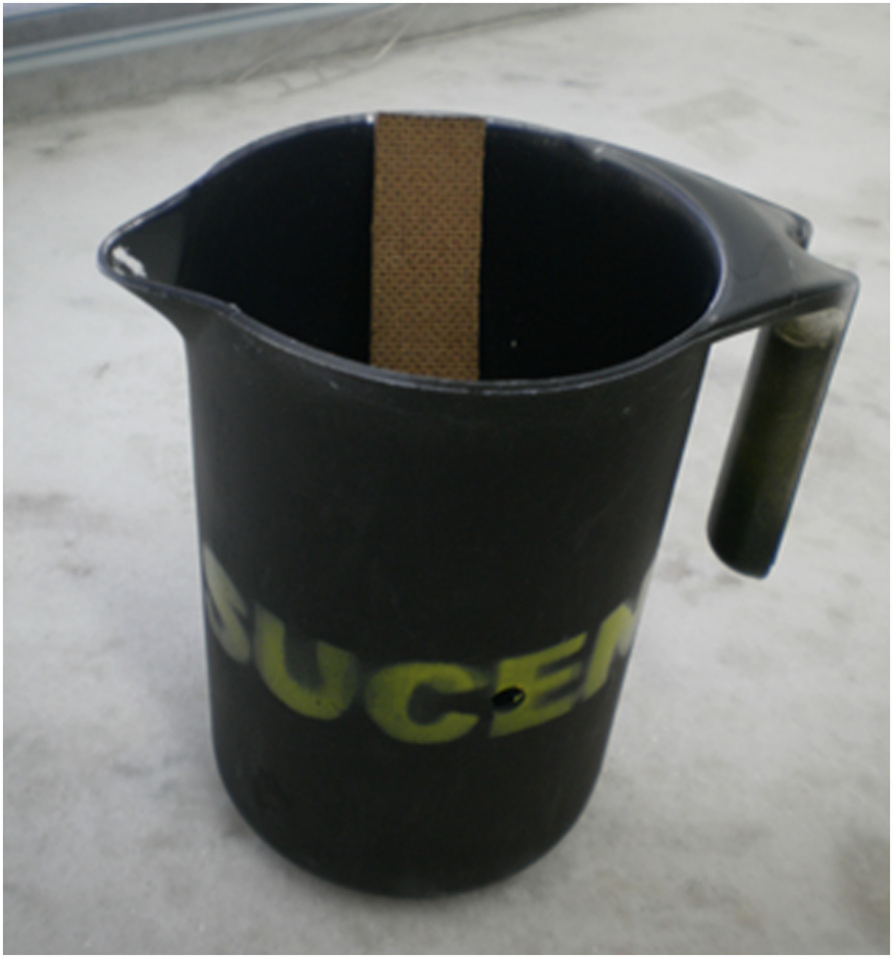
llustrative photo of the ovitrap with oviposition substratum.

The trap consists of a black plastic material (10.5 cm in diameter and 15 cm in height) filled with 450 ml of tap water and 50 ml of hay infusion [27]. A removable pressed wood pallet (Eucatex®; 15 cm × 2.5 cm) was placed within the trap as an oviposition substrate (Figure 2). The traps remained exposed for four consecutive days each month [28], from February 2011 to February 2012. Following each exposure period, the pallets were collected, and the attached eggs were counted and recorded. To ensure embryogenesis, the pallets were kept on the bench for six days at room temperature and relative humidity. Subsequently, the eggs were immersed in running water in plastic cups (9 cm × 11 cm × 6 cm), supplemented with a portion of Goldfish® feed, and covered in film. At the L3 or L4 stage, the larvae were fixed in 70% ethanol and identified [29].

### Data analyses

The measures of infestation by populations of *Ae. aegypti* and *Ae. albopictus* eggs were expressed using the positive ovitrap index (POI; frequency of positive traps) and mean eggs per trap (MET).

Spearman’s rank correlation coefficient was used to evaluate the association between the POI and MET of *Ae*. (*Ste*.) spp.

The Kruskal-Wallis test and the Dwass-Steel-Critchlow-Fligner (DSCF) test [30] were used to evaluate the POI and MET monthly distributions according to the environment.

Spearman’s rank correlation coefficient and simple linear regression were used to determine the association between the meteorological variables of minimum average, maximum average, and average temperatures; the total and average rainfall during the four days of trap exposure; and the number of traps with eggs and the egg count according to the species and environment.

Spearman’s rank correlation coefficient was used to express the correlation between the rainfall accumulated during the least rainy months (February and from May to October) and during the rainiest months (March, April, and from November to January) and the egg count during the respective periods.

Abiotic data regarding temperature and rainfall were collected from the Center of Integrated Agrometeorological Data (Centro integrado de informações agrometeorológicas, CIIAGRO) of the Department of Agriculture, Livestock, and Food Supply of the State of Sao Paulo Government (Secretaria de Agricultura e Abastecimento do Governo do Estado de São Paulo) of the São Sebastião municipal station.

## Results

In total, 1,040 traps were installed and 1028 recovered, and 255 (24.81%) traps showed the presence of *Ae*. (*Stegomyia*) spp. eggs, including 182 (71.37%) positive traps in the peridomiciliary premises and 73 (28.63%) positive traps in the intradomiciliary premises, following examination. Of the total 20,264 eggs, 16,217 (80.00%) were recovered in peridomiciliary premises, and 4,047 (20.00%) were recovered in intradomiciliary premises. The immersion of all of these eggs enabled the identification of both species, namely, *Ae. aegypti* and *Ae. albopictus*.

Of the total positive traps (n = 255), 190 (74.51%) corresponded to the above-mentioned species, including 89.50% (n = 170) for *Ae. aegypti* and 10.50% (n = 20) for *Ae. albopictus*. The distribution of these traps, according to the peri- and intradomiciliary environments, corresponded to 74.70% and 25.30% for *Ae. aegypti* and 75.00% and 25.00% for *Ae. albopictus,* respectively.

Of the remaining 65 (25,49%) positive traps, 30 (11.76%) presented the co-occurrence of *Ae. aegypti* and *Ae. albopictus* species, and 35 (13.73%) exhibited no larval hatching. The trap data of the species co-occurrence is included in an ongoing study and is not the focus of the present study.

The 190 pallets with single-species hatching of either *Ae. aegypti* or *Ae. albopictus* exhibited combined larval hatching of 59.53% (n = 8.851) of the 14,868 eggs collected. Eight thousand and fifty-eight larvae were identified in traps with *Ae. aegypti*, with 78.00% in peridomiciliary premises and 22.00% in intradomiciliary premises. In total, 793 hatched larvae were found in traps with *Ae. albopictus*, including 80.00% and 20.00% in the peri- and intradomiciliary premises, respectively.

Table 1 presents the POI and MET comparisons. The statistical analysis comparing the POI between the environments and species revealed significant differences (H = 30.51, p = 0.00). The POI of *Ae. aegypti* was noticeably higher than the POI of *Ae. albopictus* in both environments (peridomiciliary p = 0.00 and intradomiciliary p = 0.00). The POI of *Ae. aegypti* in the peridomiciliary premises was noticeably higher than in the intradomiciliary premises (p = 0.03). In contrast, the POI of *Ae. albopictus* was similar in both domiciliary environments (p = 0.33).

**Table 1:** **Comparisons of POI and MET between the *Ae. aegypti *** **and *Ae. albopictus *** **species according to the peri- and intradomiciliary environments in the municipality of São Sebastião, from February 2011 to February 2012**

**Species**	**Environment**	**POI**			**MET**		
		**Mean ± Standard deviation**	**p**	**Ratio**	**Mean ± Standard deviation**	**p**	**Ratio**
*Ae. aegypti*	Peridomiciliary	24,52 ± 4,18	0,03		12,12 ± 2,98	0,03	
x	x	x		2,92:1,00	x		3,55:1,00
*Ae. aegypti*	Intradomiciliary	8,41 ± 1,76			3,41 ± 0,94		
*Ae. albopictus*	Peridomiciliary	2,96 ± 0,91	0,33		1,27 ± 0,48	0,57	
x	x	x		3,18:1,00	x		4,38:1,00
*Ae. albopictus*	Intradomiciliary	0,93 ± 0,44			0,29 ± 0,16		
*Ae. aegypti*	Peridomiciliary	24,52 ± 4,18	0,00		12,12 ± 2,98	0,00	
x	x	x		8,28:1,00	x		9,54:1,00
*Ae. albopictus*	Peridomiciliary	2,96 ± 0,91			1,27 ± 0,48		
*Ae. aegypti*	Intradomiciliary	8,41 ± 1,76	0,00		3,41 ± 0,94	0,01	
x	x	x		9,04:1,00	x		11,76:1,00
*Ae. albopictus*	Intradomiciliary	0,93 ± 0,44			0,29 ± 0,16		

The mean eggs per trap (MET) and per environment presented statistically significant differences between the species and the environments (H = 27.60, p = 0.00). The multiple comparison demonstrated that the MET of *Ae. aegypti* was significantly greater than the MET of *Ae. albopictus* in the peridomiciliary (p = 0.00) and intradomiciliary (p = 0.01) premises.

The peridomiciliary environment had the highest MET of *Ae. aegypti* (p = 0.03), whereas no statistically significant difference (p = 0.57) was found between environments for *Ae. albopictus*. Table 1 lists the ratios of these species per environment. The POI and MET of *Ae. aegypti* were approximately three times higher in peridomiciliary than in intradomiciliary premises. Similar values of the same variables were found for the *Ae. albopictus* species but were not significant. The ratio between species revealed that the POI of *Ae. aegypti* was eight times higher than the POI of *Ae. albopictus* in the peridomiciliary premises and nine times higher in the intradomiciliary premises. Similar results were found for the MET, albeit with greater values: 9.50 times higher in the peridomiciliary premises and 11.80 times higher in the intradomiciliary premises.

The ratio of *Ae. aegypti* eggs was 3.70 in peridomiciliary environments and 1.00 in intradomiciliary environments, whereas the ratio of *Ae. albopictus* eggs was 4.05 in peridomiciliary environments and 1.00 in intradomiciliary environments.

Table 2 presents the analysis of Spearman's rank correlation coefficient and simple linear regression calculations between the number of traps with eggs and the egg count per species and environment in relation to the minimum, maximum, and average temperatures. A positive correlation was found for the number of traps with *Ae. aegypti* and the egg count with the minimum, maximum, and average temperatures in the peridomiciliary premises, and a statistically significant correlation with the maximum and average temperatures was found in the intradomiciliary premises. Regarding *Ae. albopictus*, a positive correlation was found between the maximum temperature and the number of traps with eggs in the peridomiciliary premises. No other correlations of these species were significant.

**Table 2:** **Spearman’s rank correlation coefficient and simple linear regression between the temperature and rainfall and the number of traps with eggs and egg count per trap, according to the *Ae. aegypti *** **and *Ae. albopictus *** **species and peri- and intradomiciliary environments in the municipality of São Sebastião, from February 2011 to February 2012**

**Number of traps with eggs and the egg count**	**Meteorological variables**	***Ae. aegypti***			***Ae. albopictus***		
		**r**	**r**^**2**^	**p**	**r**	**r**^**2**^	**p**
Traps peridomiciliary	Minimum T°C	0.67	0.45	0.01	0.38	0.14	0.20
	Maximum T°C	0.76	0.58	0.00	0.58	0.33	0.04
	T°C average	0.76	0.58	0.00	0.52	0.27	0.07
	Total rainfall	0.49	0.24	0.09	-0.16	0.02	0.61
	Average rainfall	0.49	0.24	0.09	-0.16	0.02	0.61
Traps intradomiciliary	Minimum T°C	0.34	0.12	0.25	0.34	0.11	0.26
	Maximum T°C	0.69	0.47	0.01	0.28	0.08	0.36
	Average T°C	0.57	0.32	0.04	0.32	0.10	0.29
	Total rainfall	0.15	0.02	0.61	0.49	0.24	0.09
	Average rainfall	0.15	0.02	0.61	0.49	0.24	0.09
Eggs peridomiciliary	Minimum T°C	0.62	0.39	0.02	0.39	0.15	0.19
	Maximum T°C	0.67	0.44	0.01	0.52	0.27	0.07
	Average T°C	0.68	0.47	0.01	0.49	0.24	0.09
	Total rainfall	0.49	0.24	0.09	-0.24	0.06	0.43
	Average rainfall	0.49	0.24	0.09	-0.24	0.06	0.43
Eggs intradomiciliary	Minimum T°C	0.45	0.20	0.12	0.14	0.02	0.65
	Maximum T°C	0.66	0.43	0.01	0.19	0.04	0.52
	Average T°C	0.60	0.36	0.03	0.18	0.03	0.55
	Total rainfall	0.26	0.07	0.38	0.26	0.07	0.40
	Average rainfall	0.26	0.07	0.38	0.26	0.07	0.40

The presence of *Ae. albopictus* during the study period was small, although this species occurred in both domiciliary environments, either simultaneously or alternating between them. The absence of *Ae. aegypti* eggs in the traps occurred in September and only in the intradomiciliary premises.

**Figure 3:**
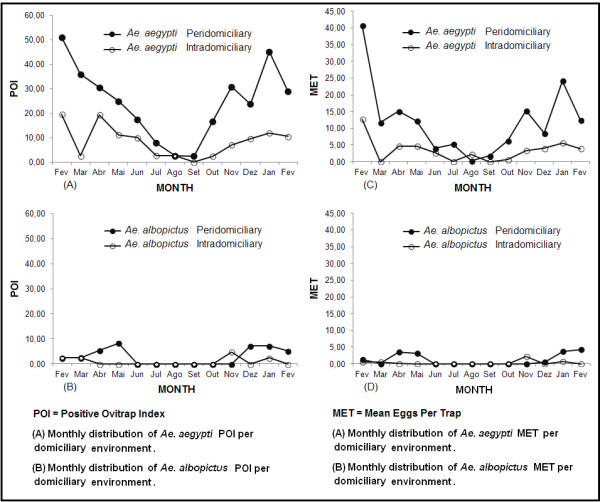
**Monthly distribution of POI and MET according to the *Ae. aegypti *** **and *Ae. albopictus *** **species.**

Figure 3 displays the POI monthly distribution of *Ae. aegypti* (A) and the POI of *Ae. albopictus* (B) per domiciliary environment. The presence of *Ae. aegypti* in peridomiciliary environments was recorded from the first to the last collection, with two significant peaks in February 2011 (51.22%) and January 2012 (45.24%). A decrease in the values was noted from March (35.90%), with the lowest index in September (2.50%), which then increased after October (16.67%).

The absence of *Ae. albopictus* was noted from June to November in peridomiciliary premises, from April to October in intradomiciliary premises, and subsequently in December and February. The peaks of this species in the peridomiciliary premises were recorded in May, December, and January. Conversely, this species peaked in the intradomiciliary premises in November.

The egg density, expressed by the MET, ranged monthly for both species, with the lowest mean of *Ae. aegypti* eggs per trap (0.21) recorded in August and the highest mean (40.66) recorded in February 2011 in the peridomiciliary premises. The highest recorded value in the intradomiciliary premises (12.66) occurred in February 2011 (Figure 3C).

The highest MET of *Ae. albopictus* in peridomiciliary premises was recorded in February 2012 (4.26) and in intradomiciliary premises in November 2011 (2.10) (Figure 3D).

**Figure 4:**
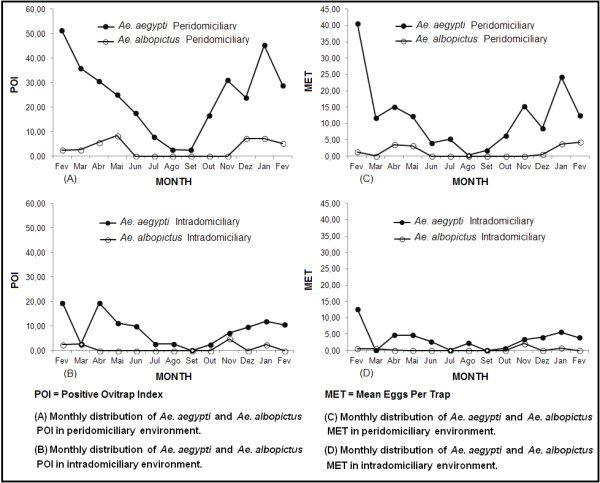
**Monthly distribution of POI and MET of *Ae. aegypti *** **compared to those of *Ae. albopictus.***

The comparison between the monthly distributions of the *Ae. aegypti* POI and the *Ae. albopictus* POI in the same domiciliary environment is presented in Figure 4 A, B. A constant and high positivity of *Ae. aegypti* was noted in the peridomiciliary premises, compared with *Ae. albopictus.* A decreased positivity of *Ae. aegypti* was noted in months where *Ae. albopictus* was absent. The intradomiciliary environment had the lowest positivity for both species.

The MET monthly distribution of *Ae. aegypti* and *Ae. albopictus* recorded peaks in the same months as those showing trap positivity for both species (Figure 4, C and D).

Spearman’s rank correlation and simple linear regression analyses between the temperature and the number of traps with eggs of both species per environment yielded significant values (Table 2). No statistically significant correlation was found between the rainfall accumulated during the least rainy months (February and May to October; r = 0.29; p = 0.49) and the rainiest months (March, April, and November to January; r = 0.60; p = 0.28) and the mean egg count of the respective periods.

Spearman’s rank correlation was determined between the POI and MET of *Ae*. (*Ste*) spp. (r = 0.94, p = 0.00).

## Discussion

The research on *Stegomyia* eggs conducted in an urban, residential area of the municipality of São Sebastião from February 2011 to February 2012 revealed that *Ae. aegypti* and *Ae. albopictus* are domesticated and able to lay eggs in peri- and intradomiciliary premises; however, *Ae. aegypti* was present in all 13 collections performed, whereas *Ae. albopictus* was present in a reduced number of collections.

The higher positivity of traps with *Ae. aegypti* showed this species’ predominance over *Ae. albopictus* in peri- and intradomiciliary environments. Such results corroborate those recorded by Lim *et al*. [16] in two Malaysian fishing towns. Those authors reported that the sparse vegetation found in their study environment might have affected the data found. In the present study, this environment was considered the peridomiciliary setting.

The process of geographic expansion of these species from 1996 to 2000 in the study municipality exhibited a trend of prevalence of *Ae. aegypti* larvae over *Ae. albopictus* larvae*.* Such findings were recorded in artificial breeding grounds, which were deemed most likely responsible for the production and maintenance of *Stegomyia* populations at that time [22].

The MET of *Ae. aegypti* in this study was higher than the MET of *Ae. albopictus* in both domiciliary environments studied. This result was similar to the findings of other authors [15,16,18]. The superiority of *Ae. aegypti* in urban habitats in general is attributed to its high anthropophily and domesticity [4].

Comparisons of the POI of *Ae. aegypti* between peri- and intradomiciliary environments indicated the predominance of this species in the peridomicile. These results corroborate the findings of Dibo *et al*. [19] in their investigation of the best location to install *Ae. aegypti* traps, which was conducted in a town in São Paulo State, Brazil.

The data reported herein characterized the peridomiciliary environment as primordial for gravid *Ae. aegypti* females in the selection of spaces for oviposition because the trap was placed both in peri- and intradomiciliary environments. Although females entered the homes to feed and rest, the gravid females found more suitable conditions for laying the eggs in the peridomiciliary environment. Chiaravalloti *et al*. [31] determined the relationship of this species with containers located in peri- and intradomiciliary premises, demonstrating that *Ae. aegypti* is more associated with the vicinity of the house, whereas *Ae. albopictus* occupies natural and disposable breeding grounds in sites farther away from peridomiciliary premises.

The present study revealed similar distribution and abundance patterns for *Ae. albopictus* in both environments studied. This result coincides with the characteristics typical of this species, which preferentially occupies peridomiciliary premises, as reported by Foo *et al.*[32]. It is noteworthy that the trap sites in the peridomiciliary premises were generally closer to the residences. Dieng *et al*. [33] suggested that this finding demonstrates the adaptative behavior of *Ae. albopictus* to intradomiciliary premises, which may cause an increase in the vectorial capacity of the species. Such records have ecological implications of epidemiological impact because they express the proximity of this species to humans, given their proficiency in transmitting different types of viruses, because this species is present in urban, peri-urban, and wild environments [34,35].

Lim *et al.*[16] demonstrated a higher positivity of traps with *Ae. albopictus* in peridomiciles, and Norzahira *et al*. [17] found a higher density and increased presence of *Ae. albopictus* in that type of domiciliary environment.

The number of positive traps was three times higher (similar to the MET, which was four times higher) in the peridomiciliary environments than in the intradomiciliary environments; these values were always expressed in small numbers throughout the study, which might have affected the statistical analysis. The low POI and MET values of *Ae. albopictus* and its similar distribution between environments may result from larval competition with *Ae. aegypti*, which is an interaction supposedly occurring in local breeding grounds [36].

In contrast with São Sebastião, the dominance pattern of these species, as noted by Norzahira *et al*. [17] in Pahang, Malaysia, indicated that populations of *Ae. albopictus* were predominant over those of *Ae. aegypti* in both domiciliary environments. Those authors recorded a higher density of *Ae. albopictus* in peridomiciliary environments. *Ae. aegypti* presented a stable population in the study area of the above-mentioned investigation, although in a disadvantaged situation.

Rozilawati *et al*. [15] found a higher abundance of *Ae. albopictus* over *Ae. aegypti* when studying their seasonal abundance in exclusively peridomiciliary environments in Penang, Malaysia*.* The authors attributed the results to the installation of ovitraps only in peridomiciliary premises.

As suggested by various authors, the lower frequency of *Ae. albopictus* eggs in an urban space may result from the wild characteristics maintained by the species, which determine its superiority in areas of significant plant cover [4,5,16]. In São Sebastião, the urban characteristics apparently favor *Ae. aegypti*. The lack of statistically significant differences for the *Ae. albopictus* indicators does not mean that this species is less attracted to a given domiciliary environment because the number of specimens recorded in peridomiciliary premises was higher than that in intradomiciliary premises. Therefore, it is legitimate to ensure the existence of different patterns of habitat occupation exercised by *Ae. aegypti* and *Ae. albopictus*. Reiter [20] reported that the oviposition behavior in the field might vary significantly both between species and within a species, albeit in different regions. Such differences are apparently related to environmental heterogeneity and the impact that human activities have on these mosquitoes [5,7,37,38].

Research studies conducted in Selangor, Malaysia, revealed no changes in the density of *Ae. aegypti* in domiciliary environments [16,18]. These similarities were interpreted as being caused by changes in the occupation pattern of *Ae. aegypti* in that region [16].

The present study demonstrated the effect of temperature on oviposition activities because a positive association was found between the temperature and the number of traps for both species and the number of *Ae. aegypti* specimens. The minimum, average, and maximum temperatures exhibited a positive association with the oviposition of *Ae. aegypti* in the peridomiciliary environment and with the average and maximum temperatures in the intradomiciliary environment. Within the residence, the minimum temperature may remain higher than in the peridomiciliary environment, consequently affecting the vector behavior, which requires additional studies. The association between the oviposition activity of *Ae. aegypti* and the temperature was clear, supported by the reductions in POI and MET that were recorded during the period from June to September, when the temperature decreased and the maximum temperature did not surpass 23.06°C. The statistically significant association found between temperature and trap positivity and the mean egg count was reported by Dibo *et al*. [19].

Azil *et al*. [7] noted that the minimum and daily average temperatures were the most significant factors associated with short- and long-term vector abundance and suggested the prospective use of meteorological variables in predicting changes in the dengue-virus vector abundance.

The lack of association between the rainfall and the trap positivity and egg density for both species, as found in the present study, may be related to local climate characteristics, namely, a tropical rainy climate with frequent rainfall and no defined dry season. Notably, the trap is an attractive breeding ground during the period of exposure. Accordingly, the lack of correlation between the rainfall accumulated during the least rainy months and during the rainiest months and the egg count recorded during the respective periods has a timely explanation. Mogi *et al*. [39] and Miyasaki *et al*. [40] reported that rainfall is a key meteorological variable for regulating vector populations using breeding grounds located in peridomiciliary premises, especially in regions with a defined climate season, which does not occur in São Sebastião.

Gravid *Ae. aegypti* females presented a temporal pattern of permanent oviposition activity. Overall, the largest increases in trap positivity and egg density occurred during the months of higher temperatures, that is, from October (average temperature of 24.32°C), stabilizing until February (minimum temperature of 20.14°C). There was a decrease in oviposition activity after February, which continued until September, when the maximum temperature decreased to 21.48°C. Such a seasonal profile of the *Ae. aegypti* population was previously found in the same study municipality using the house, container, and Breteau indices of infestation [22].

According to Vezzani *et al*. [41], the seasonal fluctuation in *Ae. aegypti* abundance enables the identification of the most appropriate moment for control intervention in the field. The highest values of the POI and MET of *Ae. aegypti* were recorded during the months with the highest temperatures, which was the most important environmental factor in vector biology at the local level.

The number of eggs collected using ovitraps from June to October, albeit small, suggests the existence of permanent breeding grounds in the area, which favors the maintenance of *Ae. aegypti* populations during the period that is least favorable to the vector, although at a low density. Souza *et al.*[42] reported that *Ae. aegypti* does not exclusively depend on the breeding grounds that emerge during the rainy period, thereby enabling this mosquito to maintain its life cycle during the dry period. For this purpose, permanent artificial breeding grounds are used, and although they are at a low density, they are sufficient to sustain transmission.

The presence of *Ae. albopictus*, in turn, was reduced, albeit with more expressive numbers, in the collections performed in May, December, and January. These last two months are among the months with highest infestation by this species, demonstrating a close relationship between its abundance and higher rainfall rates [43,44]. Different authors reported that seasonal changes in the oviposition of these species result from changes in climate conditions and the availability of oviposition sites [1,44].

The strong correlation between the POI and MET of *Ae.* (*Ste*) spp. found in the present study may be considered an association predictive of abundance and frequency of these *Stegomyia* spp [19,39]. This result would imply waiving the egg count, indicating the moment for triggering the control measures. The use of this indicator may also contribute to reducing the *Ae. aegypti* numbers when the vector population increases. Such implications were indicated by Mogi *et al.*[45], Dibo *et al.*[19], and Regis *et al*. [46], corroborating the importance of using ovitraps.

Regarding *Ae. aegypti*, the peaks of POI and MET overlapped in both environments studied. Conversely, *Ae. albopictus* exhibited a similar behavior in the intradomiciliary premises, whereas the POI peaks in the peridomiciliary premises were accompanied by a reduction in MET, and the decrease in POI was accompanied by a trend toward an increased MET. The curve of *Ae. albopictus* might have resulted from the reduced number of mosquitoes existing in the area, thereby requiring a larger sample than that used in the present study.

The use of ovitraps exhibited the seasonal variation of the *Ae. aegypti* and *Ae. albopictus* species in an urban area of São Sebastião. However, the population dynamics of these mosquitoes also results from other bioecological determinants [47], which may represent a limitation of the present study.

In this investigation, using 1,028 ovitraps, 20,264 eggs were collected from urban residences, with a mean of 20 eggs per trap. This result, combined with other control methods, may represent another strategy for reducing the population of *Ae. aegypti* through the large-scale removal of eggs, as suggested by Regis *et al*. [46].

The egg collection method was conclusively effective in assessing the distribution and abundance of *Ae. aegypti* and *Ae. Albopictus,* based on ovitraps as an attractive solution and as a possible control tool for reducing the *Ae. aegypti* population.

## Conclusions

The trap positivity and mean egg count of *Ae. aegypti* were clearly higher than those of *Ae. albopictus*. The peridomiciliary premise was the primary environment in which gravid *Ae. aegypti* females selected their oviposition site. The use of this method for vector surveillance and control activities is reiterated.

## Competing interests

The authors declare they have no competing interests and the sponsors had no role in the study design, data collection and analysis, decision to publish, or preparation of the manuscript: Study of the distribution and abundance of the eggs of *Aedes aegypti* and *Aedes albo*pictus according to the habitat and meteorological variables, municipality of São Sebastião, São Paulo, Brazil.

## Authors’ contributions

LLNS: Conceived of the study, have made the conception and design acquisition of data, have been involved in drafting the manuscript and coordinated fieldwork. GRAMM: Guiding the study product thesis, has been involved in the realization revising it critically for important intellectual content participated in the preparation and writing of the manuscript. JCV: Has made substantial contributions to studies conducted analysis and interpretation of data. APL: Participated in the coordination of the work of data collection, carried out sample collection in the field and. carried out the larvae diagnostic work. MBA: Have been involved in the realization of the sample design, participated in the coordination of the work of data collection and helped to draft the manuscript. GLB: Helped to draft the manuscript and have been involved in the revised. VRB: Helped to draft the manuscript and have been involved in the revised. VLCL: Research project coordenator of which this study is a part of. Discussed data and made revising it critically for important intellectual content. All authors read and approved the final manuscript.
